# A Sub-Nanostructural Transformable Nanozyme for Tumor Photocatalytic Therapy

**DOI:** 10.1007/s40820-022-00848-y

**Published:** 2022-04-12

**Authors:** Xi Hu, Nan Wang, Xia Guo, Zeyu Liang, Heng Sun, Hongwei Liao, Fan Xia, Yunan Guan, Jiyoung Lee, Daishun Ling, Fangyuan Li

**Affiliations:** 1 Institute of Pharmaceutics, Hangzhou Institute of Innovative Medicine, College of Pharmaceutical Sciences, Zhejiang University, Hangzhou, 310058 People’s Republic of China; 2 Frontiers Science Center for Transformative Molecules, State Key Laboratory of Oncogenes and Related Genes, School of Chemistry and Chemical Engineering, National Center for Translational Medicine, Shanghai Jiao Tong University, Shanghai, 200240 People’s Republic of China; 3 Department of Clinical Pharmacy, The First Affiliated Hospital, Zhejiang University School of Medicine, Hangzhou, 310003 People’s Republic of China; 4 WLA Laboratories, Shanghai, 201203 People’s Republic of China; 5 Department of Biomedical-Chemical Engineering, The Catholic University of Korea, 43 Jibong-ro, Wonmi-gu, Bucheon-Si, Gyeonggi-do 14662 Republic of Korea

**Keywords:** Nanozymes, Sub-nanostructural transformation, Catalytic activity, Reactive oxygen species, Photocatalytic therapy

## Abstract

The structural change-mediated catalytic activity regulation plays a significant role in the biological functions of natural enzymes. However, there is virtually no artificial nanozyme reported that can achieve natural enzyme-like stringent spatiotemporal structure-based catalytic activity regulation. Here, we report a sub-nanostructural transformable gold@ceria (STGC-PEG) nanozyme that performs tunable catalytic activities via near-infrared (NIR) light-mediated sub-nanostructural transformation. The gold core in STGC-PEG can generate energetic hot electrons upon NIR irradiation, wherein an internal sub-nanostructural transformation is initiated by the conversion between CeO_2_ and electron-rich state of CeO_2−*x*_, and active oxygen vacancies generation via the hot-electron injection. Interestingly, the sub-nanostructural transformation of STGC-PEG enhances peroxidase-like activity and unprecedentedly activates plasmon-promoted oxidase-like activity, allowing highly efficient low-power NIR light (50 mW cm^−2^)-activated photocatalytic therapy of tumors. Our atomic-level design and fabrication provide a platform to precisely regulate the catalytic activities of nanozymes via a light-mediated sub-nanostructural transformation, approaching natural enzyme-like activity control in complex living systems.

**Supplementary Information:**

The online version contains supplementary material available at 10.1007/s40820-022-00848-y.

## Highlights

- An internal sub-nanostructural transformation of sub-nanostructural transformable gold@ceria (STGC-PEG) is initiated by the conversion between CeO_2_ and electron-rich state of CeO_2−x_, and active oxygen vacancies generation via the hot-electron injection from gold to ceria.
- The sub-nanostructural transformation of STGC-PEG enhances the peroxidase-like activity and activates the plasmon-promoted oxidase-like activity, resulting in an augmented reactive oxygen species output.
- STGC-PEG successfully achieves excellent low power (50 mW cm^−2^) near-infrared light-activated photocatalytic ablation of tumors in vivo.

**Supplementary Information:**

The online version contains supplementary material available at 10.1007/s40820-022-00848-y.

## Graphical abstract

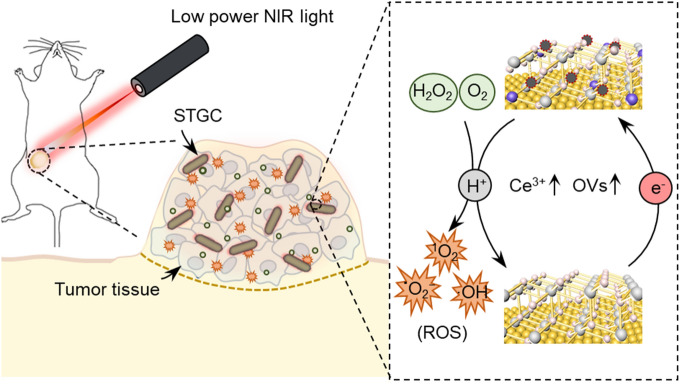

## Introduction

Enzymes, as powerful biocatalysts, have been widely used in biomedical research due to their high catalytic activities and substrate specificity [1, 2]. Though promising, the difficulties in storage and recycling, low chemical durability, and high cost of natural enzymes have limited their broad applications [3]. In recent years, artificial nanozymes with natural enzyme-mimetic activities have been developed [4–10], which attracted remarkable attention for various biomedical applications including biosensing, cytoprotection, tumor therapy, etc. [11–14]. However, different from natural enzymes, these artificial nanozymes generally lack stringent spatiotemporal structure regulation for a controllable catalytic activity in vivo, thus being undeniably far less efficient than natural enzymes [3, 15]. Up to date, tremendous efforts have been devoted to regulating the catalytic activities of nanozymes [11, 15–21]. The introduction of photo-sensitive functional ligands represents a major approach [11, 15–17]. For instance, based on the ultraviolet (UV) light-driven trans–cis isomerization of the azobenzene groups, imparting nanozymes with azobenzene containing surface ligands could selectively turn on their catalytic activity in a light-controlled manner [11, 15–17]. Unfortunately, the limited tissue penetration depth and genotoxicity of UV light as well as indirect structure regulation-based activity control restrict their further in vivo applications [22]. Self-assembly/disassembly remains another approach to tuning the enzymatic activities of nanozymes via controlled sequestering/exposing of their surface catalytic sites, which, however, lacks the spatiotemporal controllability in harsh biological microenvironments [11, 17–20].

In living systems, the regulation of catalytic activities of natural enzymes generally depends on the dynamic rearrangement of their intrinsic structures [23–25]. For instance, chymotrypsinogen can be activated by trypsin-mediated cleavage of peptide bonds between Arg15 and Ile16 [26], and DNA photolyase would undergo a concerted structural change during photoactivation to initiate the repair of the impaired duplex [27]. The structural reconfiguration-medicated regulation of enzymatic activities is essential to the coordination of numerous biochemical events in living systems [24, 28, 29], which may represent the ultimate and yet to be implemented strategy to endow nanozymes with natural enzyme-like regulatability.

Inspired by nature, we herein propose a photon-driven sub-nanostructural transformable nanozyme that performs tunable catalytic activities via intrinsic sub-nanostructural transformation. Plasmonic metal nanomaterials (e.g., Au, Ag nanostructures) have a unique surface plasmon resonance (SPR) effect and can generate energetic hot electrons upon resonant light excitation [30–33]. Particularly, when coupled with semiconducting nanomaterials (e.g., CeO_2_, TiO_2_ nanostructures), the electronic properties of the conjoined nanomaterials can be further exploited through the direct plasmon-excited electron transfer, thus boosting their photocatalytic performances [34–36]. Consequently, the designed integration of plasmonic metal/semiconductor nanostructures is anticipated to achieve the photon-driven sub-nanostructural transformation via regulating their electronic properties to initiate the local atomic reconstruction [37, 38], so as to facilitate the catalytic activity regulation of nanozymes.

**Fig. 1:**
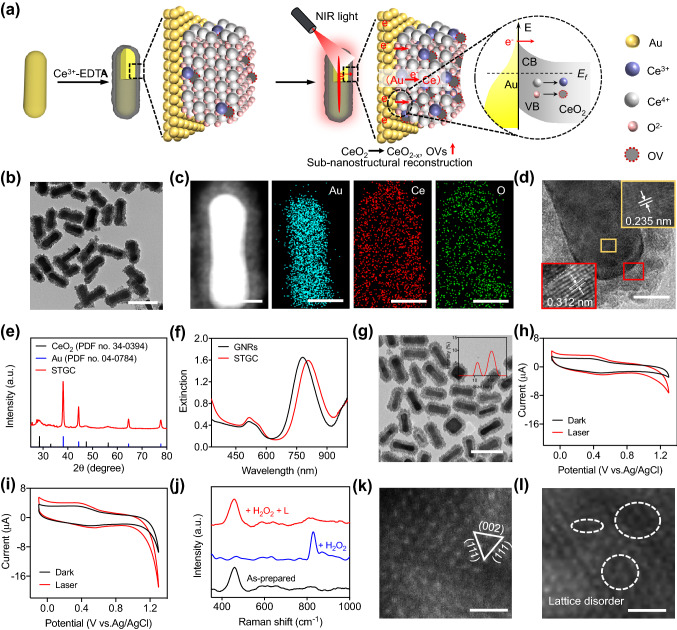
Design and characterization of photon-driven sub-nanostructural transformable nanozymes. **a** Schematic illustration of the design and photon-driven sub-nanostructural transformation of STGC through the direct electron transfer. Once triggered by near-infrared (NIR) irradiation, plasmon-excited hot electrons directly transfer from Au to CeO_2_, thus converting CeO_2_ to electron-rich state of CeO_2−*x*_, and producing active oxygen vacancies (OVs) to dynamically reconstruct the sub-nanostructure of STGC. **b** TEM image of STGC. Scale bar, 100 nm. **c** HAADF-STEM image of a single STGC and the corresponding elemental mapping. Scale bar, 20 nm. **d** HRTEM images of STGC. Scale bar, 10 nm. **e** XRD pattern of STGC. **f** UV–Vis absorption of GNRs and STGC. The LSPR peak of GNRs red shifts from 781 to 807 nm after CeO_2_ coating. **g** TEM image of PEGylated STGC (STGC-PEG). Inset: hydrodynamic diameter distribution of STGC-PEG. Scale bar, 100 nm. **h, i** Cyclic voltammograms of STGC (black line) and STGC + laser (red line, 808 nm) without **(h)** and with H_2_O_2_ treatment **(i)**. **j** Raman spectra of STGC after different treatments (L denotes laser) (*λ*_ex_ = 488 nm). **k, l** HAADF-STEM images of STGC treated with H_2_O_2_ before **(k)** and after **(l)** 808 nm laser irradiation. The O-related defect induced lattice disorder is indicated by the dashed circles. Scale bar, 1 nm

To prove our concept, a sub-nanostructural transformable gold@ceria (STGC) nanozyme was synthesized by controlled assembly of ultrafine ceria nanoparticles (CeO_2_ NPs) onto the plasmonic gold nanorods (GNRs), which provides strong near-infrared (NIR) light absorption and abundant surface reactive sites for efficient catalytic reactions [39]. Unprecedentedly, once triggered by 808 nm irradiation, plasmon-excited hot electrons directly transferred from Au to CeO_2_, converting CeO_2_ to electron-rich state of CeO_2-x_ and inducing the generation of active oxygen vacancies (OVs) to dynamically reconstruct the sub-nanostructure of STGC-PEG (Fig. 1a). Such an internal sub-nanostructural transformation ingeniously regulated the peroxidase (POD)- and oxidase (OXD)-like catalytic activities of STGC-PEG for reactive oxygen species (ROS) generation, enabling highly efficient photocatalytic therapy (PCT) of tumors. The as-designed sub-nanostructural transformable STGC-PEG represents a proof-of-concept of natural enzyme-like catalytic activity regulation via allosteric nanozymes for precisely controllable ROS-based nanomedicines.

## Experimental and Calculation

### Synthesis of STGC

The CTAB-capped GNRs were synthesized by the typical seed-mediated method [40]. NaBH_4_ (10 mM, 50 µL) was injected into a mixture containing CTAB (0.1 M, 1.95 mL) and HAuCl_4_ (10 mM, 50 µL) and stirred for 2 min to obtain the seed solution, which was placed at 27 °C for 2 h before use. For the preparation of growth solution, HAuCl_4_ (10 mM, 15 mL), AgNO_3_ (4 mM, 7.5 mL), AA (0.1 M, 1.2 mL) and the seed solution (450 µL) were injected into CTAB (0.1 M, 300 mL), and the solution was stirred for 2 min and then kept undisturbed at 27 °C at least 3 h. The final product was collected by centrifugation at 10,000 rpm for 15 min and re-dispersed into deionized water (DI water) as the stock solution (0.4 nM). STGC was synthesized through a modified solvothermal method [39]. For the preparation of STGC, CTAB (0.2 M, 1.125 mL), EDTA-NH_3_ (10 mM, 0.35 mL) and CeCl_3_ (0.1 M, 0.035 mL) were sequentially added into the GNRs stock solution (8 mL). Then, the solution was kept at 90 °C for 1.5 h in an oven. The final product was centrifuged and re-dispersed into DI water (2 mL) for further use.

### ***Synthesis of Alendronate-Conjugated mPEG***_***5K***_*** (mPEG***_***5K***_***-ALN)***

mPEG_5K_-ALN was synthesized according to the reported method by Yang et al. [41]. Briefly, mPEG_5K_-COOH (500 mg), EDC (30 mg), and NHS (15 mg) were dissolved into DI water (4 mL) and stirred for 30 min. Then, alendronate sodium trihydrate (100 mg) and Na_2_CO_3_ (40 mg) were dissolved in DI water (2 mL) and added to the above solution. The reaction was carried out for 24 h, followed by dialysis and freeze-drying before collecting the final product.

### Surface Modification of STGC

STGC (70 μg mL^−1^, 2 mL), mPEG_5K_-SH (10 mg), and mPEG_5K_-ALN (30 mg) were mixed in 2 mL DI water for the ligand exchange process, and the mixture was stirred at room temperature (RT) for 24 h. The resulting PEGylated STGC was isolated via centrifugation and rinsed three times with DI water for further use.

## Results and Discussion

### Synthesis of STGC

Ultrafine CeO_2_ NPs were assembled on the surface of GNRs (Fig. S1) through a modified solvothermal method [39] to form well-dispersed STGC (Figs. 1b and S2-S3). Energy-dispersive spectrum (EDS) (Fig. S4a), line-scanning EDS (Fig. S4b), and elemental mapping (Fig. 1c) illustrate the distribution of Ce and O on the surface of GNRs. The mass ratio of Au: Ce in STGC is ~ 3: 1 as measured via inductively coupled plasma mass spectrometry (ICP-MS), where the relatively high Au-to-Ce ratio guarantees the sufficient hot electrons supply from Au and further photon-driven sub-nanostructural transformation of STGC. High-resolution transmission electron microscopy (HRTEM) (Fig. 1d) and X-ray diffraction (XRD) (Fig. 1e) pattern reveal the (111) plane of the fluorite cubic CeO_2_ phase and the (111) plane of cubic Au phase, demonstrating the poly-crystalline nature of STGC. Besides, the red-shift of longitudinal surface plasmon resonance (LSPR) peak further suggests the successful coating of CeO_2_ on GNRs, owning to the CeO_2_ coating-induced local increase of refractive index, where strong NIR adsorption endows STGC with potential photo-triggered catalytic activity [39] (Fig. 1f). Moreover, X-ray photoelectron spectroscopy (XPS) spectrum reveals the coexistence of Ce^3+^ and Ce^4+^ in STGC (Fig. S5), suggesting the presence of OVs that maintain the charge balance [5, 38]. For further biomedical applications, the STGC was modified with poly(ethylene glycol) (PEG) (Fig. 1g), and the PEGylated STGC (STGC-PEG) with an average hydrodynamic diameter of ~ 128.5 nm (Fig. 1g, inset) and a negative charge (Fig. S6), is highly stable in water (Fig. S7). The successful PEGylation was further confirmed by XPS and Fourier transform infrared (FT-IR) (Fig. S8).

### Photon-Driven Electron Transfer-Mediated Sub-Nanostructural Transformation

To verify the photon-driven electron transfer-mediated sub-nanostructural transformation of STGC, cyclic voltammetry (CV) curves of STGC were initially recorded. The symmetrical redox peaks at around + 0.51 and + 0.35 V are assigned to the redox of Ce^3+^/Ce^4+^ in CeO_2_ [42, 43]; upon NIR irradiation, a significant peak current enhancement of STGC can be observed (Fig. 1h). The electric current enhancement agrees well with the proposal that the plasmon-excited hot electrons overcome the Schottky barrier and then transfer from Au to CeO_2_ [39, 44], leading to the generation of Ce^3+^ and active OVs [42, 45, 46]. Intriguingly, the increase in electric current also occurred in H_2_O_2_-pretreated STGC (Fig. 1i). Besides, the regeneration of Ce^3+^ and OVs of CeO_2_ NPs in the presence of H_2_O_2_ would be inhibited in acidic conditions [38, 47]. Therefore, the regeneration ability of CeO_2_ NPs in STGC can be restored, benefiting from the electron transfer from Au to CeO_2_ upon laser irradiation. Consistently, the LSPR peak of STGC red-shifts (Ce^3+^ → Ce^4+^) after co-incubation with H_2_O_2_ in acidic condition [39] and returns upon laser irradiation (Fig. S9). Moreover, as shown in Raman spectra (Fig. 1j), the initial 455 cm^−1^ peak (a symmetric breathing mode of oxygen atoms surrounding Ce ions [47, 48]) diminishes and a new peak at ~ 837 cm^−1^ (O–O stretching vibration of the absorbed peroxide species [49]) is observed after co-incubation with H_2_O_2_ in acidic condition, which can also be recovered after laser (L) irradiation. Furthermore, XPS and electron spin resonance (ESR) results manifest the formation of OVs in H_2_O_2_-treated STGC after laser irradiation (Fig. S10a-b), while the XRD patterns show that the crystal planes of CeO_2_ (JCPDS No. 034-0394) do not reveal a significant change after reaction, indicating the light-mediated tunable catalytic activities are not based on the change of crystal planes (Fig. S10c). Impressively, more lattice disorder and dislocation upon laser irradiation are directly verified for H_2_O_2_-treated STGC by using atomic-resolution high-angle annular dark-field scanning transmission electron microscopy (HAADF-STEM) (Fig. 1k, l), which indicates the existence of numerous vacancies for the induction of coordinatively unsaturated metal atoms [50, 51], demonstrating the photon-driven sub-nanostructural transformation of STGC.

### Photocatalytic Performance

**Fig. 2:**
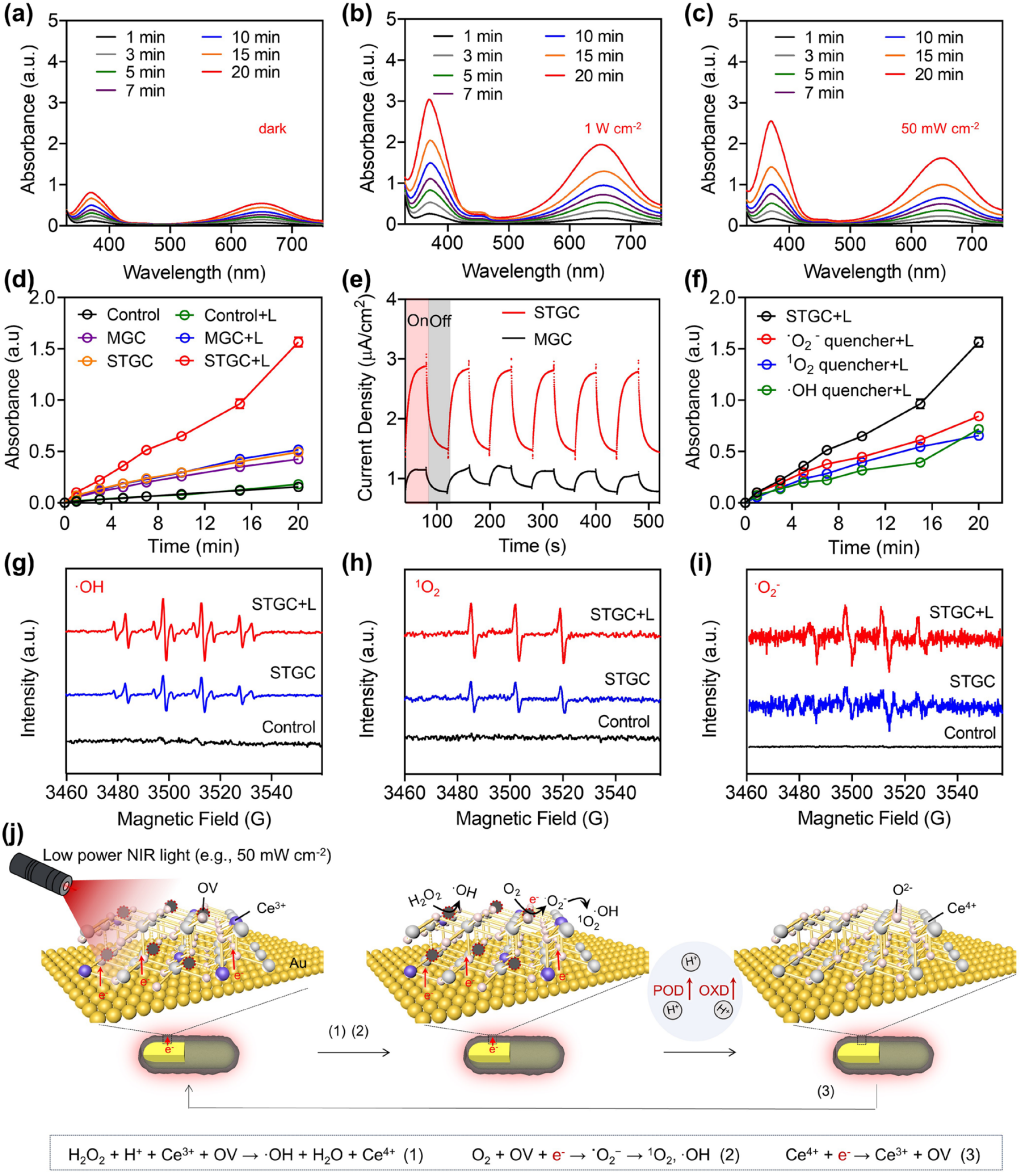
Evaluation of the photocatalytic activity regulation of photon-driven sub-nanostructural transformable nanozymes. **a**–**c** TMB absorbance spectra upon the addition of STGC-PEG and H_2_O_2_ (10 mM) without **(a)**, with 808 nm laser (1 W cm^−2^) **(b)** or low-power 808 nm laser (50 mW cm^−2^) irradiation **(c)**. STGC denotes STGC-PEG. **d** Time-course absorbance of TMB oxidation product at 650 nm upon the addition of STGC-PEG or physical mixture of GNRs-PEG and CeO_2_-PEG (MGC-PEG) with or without low-power 808 nm laser irradiation (50 mW cm^−2^) in the presence of H_2_O_2_ (10 mM). MGC denotes MGC-PEG. Data are presented as means ± s.d. (*n* = 3/group). **e** Electrochemical performances of STGC-PEG and MGC-PEG measured by *i*–*t* curve via a 808 nm laser ON–OFF switch (measured in 0.5 M H_2_SO_4_, 15 mV vs. Ag/AgCl). **f** Time-course absorbance of TMB oxidation products at 650 nm with different scavengers irradiated with low-power 808 nm laser (50 mW cm^−2^). Data are presented as means ± s.d. (*n* = 3/group). **g** ESR spectra of DMPO-OH spin adducts generated by STGC-PEG with or without low-power 808 nm laser irradiation (50 mW cm^−2^, 5 min) in the presence of H_2_O_2_ (10 mM). **h, i** ESR spectra of TEMP-^1^O_2_ **(h)**, and BMPO-˙O_2_^−^ **(i)** spin adducts generated by STGC-PEG with or without low-power 808 nm laser irradiation (50 mW cm^−2^, 5 min). **j** Schematic illustration of atomic-level photon-mediated sub-nanostructural transformation of STGC-PEG for enzymatic activity regulation. Upon NIR irradiation, the generated hot electrons from Au can convert CeO_2_ to CeO_2−*x*_ and generate active OVs, thus endowing STGC-PEG with photon-promoted peroxidase (POD)- and oxidase (OXD)-like activities

We further investigated the photocatalytic performance of STGC-PEG. The oxidative activity of STGC-PEG sharply augments under high power (1 W cm^−2^) NIR irradiation (Fig. 2a, b), which is significantly higher than that of the physical mixture of GNRs-PEG and CeO_2_-PEG (MGC-PEG) (Figs. S11 and S12). More interestingly, the low-power (50 mW cm^−2^) NIR irradiation that results in limited photothermal effect could also markedly trigger photocatalytic performance of STGC-PEG and leads to a three-fold increased oxidation of 3,3′,5,5′-tetramethylbenzidine (TMB) after 20 min (Fig. 2c, d). Besides, photocurrent measurements confirm the photon-driven electron transfer of STGC-PEG (Fig. 2e), which mediates sub-nanostructural transformation to enhance the oxidative activity. Moreover, the photon-augmented ROS are identified to be ·OH, ^1^O_2_, and ˙O_2_^−^ by using specific quenchers via UV–Vis and trapping agents via ESR analysis (Figs. 2f–i and S13), indicating photon-enhanced POD- and OXD-like activities of STGC-PEG. Specifically, extra H^+^ may retard the reduction of Ce^4+^ and thus inhibit the decomposition of absorbed H_2_O_2_ [38, 47, 52], showing a restricted POD-like activity of STGC-PEG (Fig. S14). However, upon NIR irradiation, the interband transition of Au is excited to produce electron–hole pairs, and the hot electrons transfer from excited Au to the conduction band (CB) of CeO_2_ to participate in the reduction of Ce^4+^ and produce active OVs, rebuilding the POD-like activity of STGC-PEG for H_2_O_2_ decomposition. Meanwhile, the active OVs can effectively trap the photogenerated hot electrons and O_2_ [53, 54], significantly boosting the OXD-like activity to generate toxic ˙O_2_^−^/^1^O_2_/·OH [46, 55–60] (Fig. 2j). Besides, the plasmon-excited holes on Au possess the oxidation ability and thus can drive the oxygen-evolution half-reaction (2H_2_O + 4 h^+^ → O_2_ + 4H^+^), facilitating ROS generation as well as the equilibrium of electrons on the surface of Au [44, 61–63].

### Low-Power NIR Light-Activated Tumor PCT

**Fig. 3:**
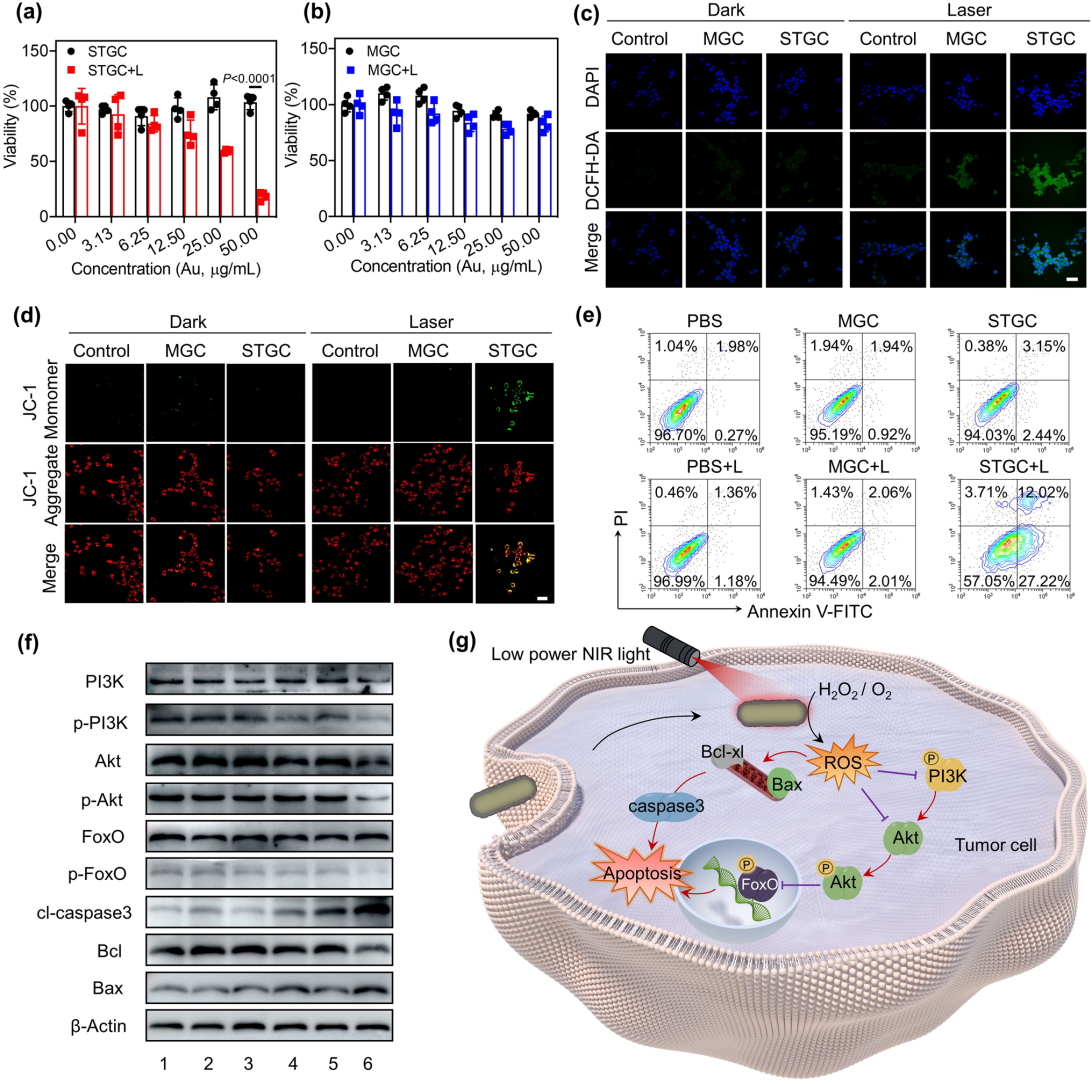
In vitro photocatalytic therapy using photon-driven sub-nanostructural transformable nanozymes. **a** Cellular toxicity profile of STGC-PEG without or with 808 nm laser irradiation (50 mW cm^−2^, 5 min). STGC denotes STGC-PEG. Data are presented as means ± s.d. (*n* = 4/group). **b** Cellular toxicity profile of MGC-PEG without or with 808 nm laser irradiation (50 mW cm^−2^, 5 min). MGC denotes MGC-PEG. Data are presented as means ± s.d. (*n* = 4/group). **c** Fluorescence microscopy images of intracellular ROS levels in MGC-PEG- and STGC-PEG-treated 4T1 tumor cells without or with 808 nm laser irradiation (50 mW cm^−2^, 5 min). Scale bar, 40 µm. **d** Fluorescence microscopy images of mitochondrial membrane potential in MGC-PEG- and STGC-PEG-treated 4T1 tumor cells without or with 808 nm laser irradiation (50 mW cm^−2^, 5 min). The mitochondrial damage is indicated by the green fluorescent signals. Scale bar, 40 µm. **e** Flow cytometry analysis of cell apoptosis of MGC-PEG- and STGC-PEG-treated groups without or with 808 nm laser irradiation (50 mW cm^−2^, 5 min). **f** Western blotting analysis of PI3k-Akt-FoXO signaling pathway and apoptosis-related proteins (1, PBS; 2, MGC-PEG; 3, STGC-PEG; 4, PBS + L; 5, MGC-PEG + L; 6, STGC-PEG + L). **g** Schematic illustration of ROS amplification and signaling pathways induced by photon-driven sub-nanostructural transformation of STGC-PEG

The photocatalytic effect of STGC-PEG was further investigated at the cellular level. STGC-PEG can be effectively internalized by 4T1 tumor cells (Figs. S15 and S16) without inducing noticeable dark cytotoxicity (Fig. 3a). Moreover, despite that GNRs-PEG show a photothermal conversion efficiency (*η*, 39.7%) comparable to that of STGC-PEG (37.2%; Fig. S17), only STGC-PEG shows significant cytotoxicity upon low-power NIR irradiation (50 mW cm^−2^), which decreases the cellular viability to 59.7% at the concentration of 25 μg mL^−1^ Au (Fig. 3a, b), with the most severe ROS generation (Fig. 3c), mitochondrial damage (Fig. 3d) and apoptosis rate of tumor cells (Fig. 3e). Besides, STGC-PEG displays a much lower cytotoxicity on human normal cell lines including hepatic cell L02 and colonic epithelial cell NCM460 even upon NIR irradiation, probably resulting from the relatively low level of H_2_O_2_ in normal cells (Fig. S18). Taken together, we conclude that upon internalization into tumor cells, the conversion from CeO_2_ to electron-rich state of CeO_2−*x*_ as well as the active OVs generation would be initiated by the hot-electron injection from gold to ceria upon low-power NIR irradiation, whereafter the dynamic sub-nanostructural transformation of STGC-PEG triggers the excess ROS generation for effective tumor cell killing.

Furthermore, we examined the changes in the molecular pathway of 4T1 tumor cells after different treatments. As shown in Fig. 3f, the levels of Akt, phosphorylated Akt (p-Akt), p-PI3K and p-FoxO, which are well implicated in breast cancer development, are downregulated by STGC-PEG upon NIR irradiation. Moreover, STGC-PEG induces the downregulation of antiapoptotic Bcl-xl, the upregulation of proapoptotic Bax and cleaved caspase-3 (cl-caspase-3) during the PCT. Consequently, we reason that the toxic ROS generated by STGC-PEG not only directly affect Bcl-2 family proteins and activate pro-apoptotic pathways [64], but also inhibit the PI3K/Akt/FoxO signaling cascade, thus promoting the tumor cell death (Fig. 3g).

**Fig. 4:**
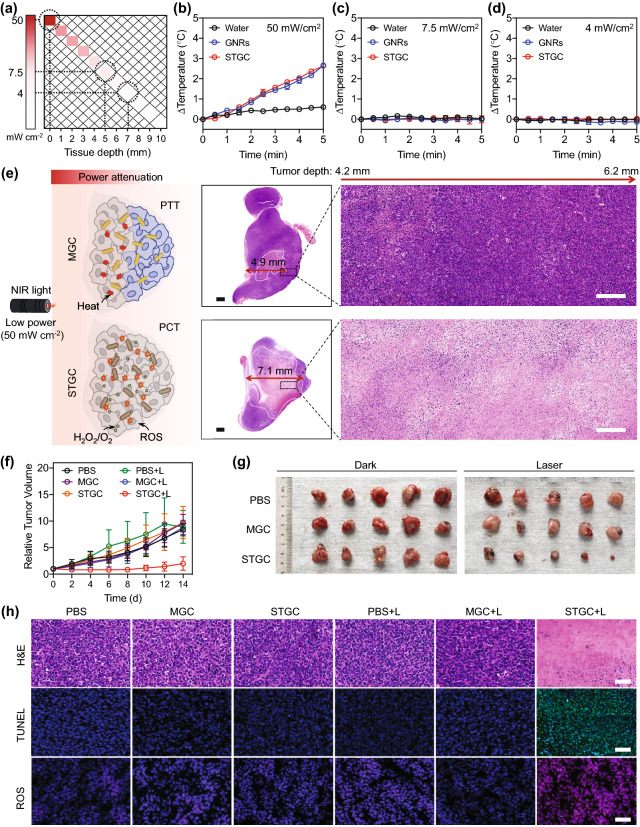
In vivo photocatalytic therapy using photon-driven sub-nanostructural transformable nanozymes. **a** The attenuation of NIR light as it passes through tissues. Data are presented as means ± s.d. (*n* = 3/group). **b-d** The change of temperature generated by water, GNRs-PEG, and STGC-PEG (0.6 mg ml^−1^, Au) under different NIR light power densities of 50 mW cm^−2^ **(b)**, 7.5 mW cm^−2^ **(c)**, and 4 mW cm^−2^ **(d)** for 5 min, which are corresponding to the power attenuation at tissue thickness of 0 mm, 5 mm, and 7 mm, respectively. STGC denotes STGC-PEG, and GNRs denote GNRs-PEG. Data are presented as means ± s.d. (*n* = 3/group). **e** The schematic illustration and H&E staining of the whole tumors of the mice after the treatments of MGC-PEG-mediated power-dependent PTT and STGC-PEG-mediated deep tumor PCT upon the attenuated laser irradiation. Scale bars, 1 mm (left) and 200 µm (right). STGC-PEG + L leads to a greater tumor necrosis depth (~ 7.1 mm) as compared to MGC-PEG + L (~ 4.9 mm). MGC denotes MGC-PEG. **f** 4T1-tumor growth curves of the mice with different treatments measured every other day. Data are presented as means ± s.d. (*n* = 5/group). **g** Photograph of tumors on the 14th day after various treatments without or with 808 nm laser irradiation (50 mW cm^−2^, 5 min). **h** H&E, TUNEL, and ROS staining of tumor tissues in different groups. STGC-PEG + L-treated group exhibits the most shrunken nucleus (in H&E staining), the highest cell apoptosis ratio (the TUNEL positive cells are indicated by the green fluorescent signals), and the highest ROS level (indicated by the increased red fluorescence intensity). Scale bar, 50 µm

Encouraged by in vitro results, we further evaluated the in vivo tumor PCT using STGC-PEG under low-power NIR irradiation (50 mW cm^−2^). Considering that high light fluency can inevitably induce the necrosis of the normal tissues [65, 66], it is appealing to achieve potent phototherapy with minimal light intensity, especially for deep-seated tumors requiring light penetration through tissue barriers that diminish light intensity at the tumor sites. Due to the power attenuation in thick biological tissues, the power density of laser sharply declines as measured by a power meter, for example, by 85.1% and 92.2% at tissue thickness of 5 mm and 7 mm, respectively (Fig. 4a–d). Interestingly, because of the sharp NIR light attenuation in deep tumor tissues, through hematoxylin and eosin (H&E) staining of the whole tumor after the laser irradiation, we find that MGC-PEG leads to limited tumor necrosis due to the power-dependent photothermal effect, while STGC-PEG induces severe tumor necrosis with a much larger area and greater depth (Fig. 4e). This finding strengthens the advantage of the low-power NIR irradiation-triggered deep tumor PCT by using STGC-PEG via the markedly high-performance photon-mediated sub-nanostructural transformation. Consequently, STGC-PEG effectively suppresses the progression of 4T1 breast cancer, while MGC-PEG shows no obvious tumor growth inhibition (Figs. 4f–g and S19). Furthermore, the ROS level in STGC-PEG + laser-treated tumor presents to be the highest among all the groups, indicating the highly efficient in situ photocatalytic reaction. H&E and terminal deoxynucleotidyl transferase dUTP nick end label (TUNEL) staining results reveal not only significantly severe cell apoptosis, but also markedly deep tumor destruction in STGC-PEG + L group (Fig. 4h). Notably, similar to control group, neither obvious body weight change (Fig. S20), nor noticeable pathological tissue damage or abnormality from the histology analysis (H&E staining) (Fig. S21) can be found in mice treated with STGC-PEG. Besides, body weights (Fig. S22), serum biochemical analysis (Fig. S23), hematological index (Fig. S24), as well as H&E staining (Fig. S25) of healthy normal BALB/c mice all demonstrate the great biocompatibility of STGC-PEG in vivo. To our best knowledge, this is the first demonstration of a nanozyme that, by virtue of the dynamic sub-nanostructural transformation-mediated catalytic activity regulation, can achieve excellent low-power NIR light-activated photocatalytic ablation of tumors in vivo; such a sub-nanostructural transformable nanozyme enabled tumor PCT is superior to conventional light-mediated therapies, such as PTT and photodynamic therapy, whose efficacy can be significantly affected by light attenuation in tissues or tumor hypoxia.

## Conclusions

We have developed a photon-driven sub-nanostructural transformable nanozyme (STGC-PEG) with light-responsive sub-nanostructural transformation that can fine-tune its catalytic activities in biological environment. Upon NIR irradiation, the plasmon-excited hot electrons transfer from Au to CeO_2_ to initiate CeO_2_–CeO_2−*x*_ conversion and generate active OVs, endowing STGC-PEG with dramatically amplified POD- and OXD-like activities. Importantly, STGC-PEG results in massive augmentation of ROS production with spatiotemporal controllability upon low-power NIR irradiation and thus potentiate the anti-tumor efficiency of PCT both in vitro and in vivo. Particularly, for the first time, STGC-PEG successfully achieves excellent low power NIR light-activated photocatalytic ablation of tumors in vivo. As a proof-of-concept, our strategy provides a new paradigm for fabricating dynamically transformable nanozyme with tunable catalytic performances in vivo for biomedical applications. Our findings may aid the future design of advanced biomimetic nanocatalysts and provide a model for approaching natural enzyme-like activity control of artificial nanozymes.

## Supplementary Information

Below is the link to the electronic supplementary material.

Supplementary file1 (DOCX 6684 kb)
