# Supplementary material for: A Sub-Nanostructural Transformable Nanozyme for Tumor Photocatalytic Therapy
Source: Nanomicro Lett. 2022 Apr 12;14:101. doi: 10.1007/s40820-022-00848-y (PMC9005554; doi:10.1007/s40820-022-00848-y)
Supplement: Supplementary file 1 — Supplementary file1 (DOCX 6684 kb) [file 40820_2022_848_MOESM1_ESM.docx]

Supporting Information for

**A Sub-Nanostructural Transformable Nanozyme for Tumor Photocatalytic Therapy**

Xi Hu^1, 2, 3, #^, Nan Wang^1, #^, Xia Guo^1, #^, Zeyu Liang^1,4, #^, Heng Sun^1^, Hongwei Liao^1^, Fan Xia^1^, Yunan Guan^1^, Jiyoung Lee^1, 5^, Daishun Ling^1, 2, 4,^ *, and Fangyuan Li^1, 4,^ *

^1^Institute of Pharmaceutics, Hangzhou Institute of Innovative Medicine, College of Pharmaceutical Sciences, Zhejiang University, Hangzhou 310058, China

^2^Frontiers Science Center for Transformative Molecules, State Key Laboratory of Oncogenes and Related Genes, School of Chemistry and Chemical Engineering, National Center for Translational Medicine, Shanghai Jiao Tong University, Shanghai 200240, China

^3^Department of Clinical Pharmacy, the First Affiliated Hospital, Zhejiang University School of Medicine, Hangzhou 310003, China

^4^WLA Laboratories, Shanghai 201203, China

^5^Department of Biomedical-Chemical Engineering, The Catholic University of Korea, 43 Jibong-ro, Wonmi-gu, Bucheon-Si, Gyeonggi-do 14662, Republic of Korea

^#^These authors contributed equally to this work.

*Corresponding author. E-mail: dsling@sjtu.edu.cn; lfy@zju.edu.cn

**Supplementary Materials**

All reagents and solvents were obtained commercially and used without further purification. Gold chloride trihydrate (HAuCl_4_·3H_2_O), sodium borohydride (NaBH_4_), ascorbic acid (AA), cerium(III) chloride heptahydrate (CeCl_3_·7H_2_O), ethylenediaminetetraacetic acid (EDTA), alendronate sodium trihydrate, 3,3’,5,5’-tetramethylbenzidine (TMB), carotene, mannite, 5,5-dimethyl-1-pyrroline-N-oxide (DMPO), 2,2,6,6-tetramethylpiperidine (TEMP), sodium acetate (NaAc), acetate were purchased from Aladdin Industrial Inc. (Shanghai, China). 5-tertbutoxycarbonyl-5methyl-1-pyrroline N-oxide (BMPO) was bought from APExBIO (Houston, USA). Superoxide dismutase (SOD) was purchased from Shanghai yuanye Bio-Technology Co. Ltd., (Shanghai, China). Cetyltrimethylammonium bromide (CTAB), silver nitrate (AgNO_3_), ammonium hydroxide (NH_3_·H_2_O), sodium carbonate (NaCO_3_), hydrogen peroxide (H_2_O_2_), dimethyl sulfoxide (DMSO) were purchased from Sinopharm Chemical Reagent Co. Ltd., (Shanghai, China). Methoxy poly(ethylene glycol)-5000-thiol (mPEG_5K_-SH), methoxy poly(ethylene glycol)-5000-carboxyl (mPEG_5K_-COOH) were purchased from Ziqi Biotechnology Co. (China).

**Characterization**

Transmission electron microscopy (TEM) images were acquired on a transmission electron microscope (Hitachi HT7700, Japan). ζ-potential and hydrodynamic size measurements were performed using a Nano ZS instrument (Malvern, U.K.). UV-Vis absorption spectra were obtained via a Shimadzu UV-2450 spectrophotometer. X-Ray diffraction (XRD) patterns were obtained via using an X-ray diffractometer (PANalytical B.V. X-pert Powder, Netherlands). X-ray photoelectron spectroscopy (XPS) was acquired by a Thermo Scientific ESCALAB 250 Xi XPS system. Raman spectra were acquired on a LabRAM HR evolution (HORIBA, France). High-angle annular dark-field scanning transmission electron microscopy (HAADF-STEM) images were obtained via using a spherical aberration-corrected Titan ChemiSTEM microscope. The molar ratios of Au and Ce in the products were determined by an X SERIES inductive coupling plasma mass spectrometer (ICP-MS) system.

**Synthesis of CeO_2_ NPs**

For the preparation of CeO_2_ NPs, CTAB (0.2 M, 1.125 ml), EDTA-NH_3_ (10 mM, 0.35 ml) and CeCl_3_ (0.1 M, 0.035 ml) were sequentially added into DI-water (8 ml). Then, the solution was kept at 90 °C for 1.5 h in an oven. The final product was centrifuged and re-dispersed into DI-water (2 ml) for further use.

**Surface Modification of GNRs and CeO_2_ NPs**

CTAB capped GNRs (30 μg ml^-1^, 2 ml) were added to the mPEG_5K_-SH solution (20 mg, 2 ml) for the ligand exchange process and the mixture was stirred at RT for 12 h, followed by centrifugation, and rinsed three times with DI-water for further use.

CeO_2_ NPs (100 μg ml^-1^, 2 ml) were added to the mPEG_5K_-ALN aqueous solution (60 mg, 2 ml) for the ligand exchange process and the reaction was carried out at RT for 24 h, followed by centrifugation, and washed three times with DI-water for further use.

**Preparation of RITC-labeled STGC-PEG**

RITC (10 mg, dissolved in 0.5 ml ethanol) was added to NH_2_-mPEG_5K_-SH water solution (200 mg, 2 ml), and the mixture was stirred at RT for 12 h, followed by the dialysis against DI-water for 48 h to obtain RITC-labeled mPEG_5K_-SH. Similarly, for the preparation of RITC-labeled mPEG_5K_-ALN, RITC (10 mg, dissolved in 0.5 ml ethanol) was added to NH_2_-mPEG_5K_-ALN water solution (200 mg, 2 ml). And the mixture was stirred at RT for 12 h, followed by the dialysis against DI-water for 48 h to obtain the product.

For the preparation of RITC-labeled STGC-PEG, STGC (70 μg ml^-1^, 2 ml), RITC-labeled mPEG_5K_-SH (10 mg) and RITC-labeled mPEG_5K_-ALN (30 mg) were mixed in 2 ml DI-water and stirred at RT for 24 h. The RITC-labeled STGC-PEG was centrifugated and rinsed three times with DI-water for further use.

**Electrochemical Analysis**

Electrochemical experiments were performed on a standard three-electrode cell connected with an electrochemical work station (CH Instruments, Inc. Shanghai), where Ag/AgCl (4 M KCl) and Pt wire were worked as the reference electrode and counter electrode, respectively. The working electrode was modified with STGC for electrochemical analysis. For preparing the working electrode, Nafion (1.5 μl) and isopropanol (10 μl) were added into the STGC (20 μl) and sonicated for 30 min. Subsequently, the mixture was poured onto the freshly polished glassy carbon electrode (0.07065 cm^2^) and dried. Similarly, GNRs-, CeO_2_-, and MGC-modified electrodes were also prepared using the same procedures. The modified working electrodes were scanned in an Ar-purged H_2_SO_4_ solution (0.5 M) with/without NIR irradiation (808 nm, 50 mW cm^-2^).

**Catalytic Activities Evaluation**

TMB was introduced to evaluate the catalytic activities of STGC-PEG with/without NIR irradiation. In detail, STGC-PEG (800 μg ml^-1^ of Ce, 10 μl) was added to HAc/NaAc buffer solution (0.2 M, 2 ml, pH = 5.0) containing H_2_O_2_ (10 mM). Then TMB (50 mM, 20 μl) was added to the above mixture to initiate the oxidizing reaction of TMB with/without 808 nm laser irradiation (50 mW cm^-2^; 1 W cm^-2^). The oxidation process of TMB was recorded at different time points by UV-vis detection at 650 nm.

**ROS Detection with TMB**

STGC-PEG (800 μg ml^-1^ of Ce, 10 μl) was added to HAc/NaAc buffer solution (0.2 M, 2 ml, pH = 5.0) containing H_2_O_2_ (10 mM). Then TMB (50 mM, 20 μl) was added to the above mixture to initiate the oxidizing reaction of TMB with/without 808 nm laser irradiation (50 mW cm^-2^; 1 W cm^-2^). In addition, different ROS quenchers were added to the solutions prior to the UV-vis detection to identify the type of ROS, wherein 100 μl mannite of 50 mM, 0.6 mg carotene, and 100 μl SOD of 4,000 unit ml^-1^, were used to identify ·OH, ^1^O_2_ and ˙O_2_^−^, respectively [1]. The oxidation process of TMB was recorded at different time points by UV-vis detection at 650 nm.

**ESR Measurements**

ESR measurements were carried out for the detection of ·OH, ^1^O_2_, and˙O_2_^−^ with 808 nm laser irradiation (50 mW cm^-2^; 1 W cm^-2^), which then were measured by the Bruker A300 EPR spectrometer.

For ·OH detection, STGC-PEG (80 μg ml^-1^ of Ce, 10 μl) was added to the mixture of DMPO aqueous solution (0.2 M, 100 μl) and HAc/NaAc solution (0.2 M, 100 μl, pH = 5.0) containing 10 mM H_2_O_2_, which were then incubated for 5 min before ESR measurement. For the laser (L)-treated group, the resulting solution was irradiated under 808 nm laser (50 mW cm^-2^; 1 W cm^-2^) for 5 min before detection.

For ^1^O_2_ detection, STGC-PEG (80 μg ml^-1^ of Ce, 10 μl) was added to the mixture of TEMP (1 M, 57 μl) in DMSO solution and HAc/NaAc solution (0.2 M, 143 μl, pH = 5.0), which were then incubated for 5 min before ESR measurement. For the L-treated group, the resulting solution was irradiated under 808 nm laser (50 mW cm^-2^; 1 W cm^-2^) for 5 min before detection.

For ˙O_2_^−^ detection, STGC-PEG (80 μg ml^-1^ of Ce, 10 μl) was added to the mixture of BMPO (25 mM, 100 μl) in HAc/NaAc solution (0.2 M, pH = 5.0) and DMSO solution (100 μl), which were then incubated for 5 min before ESR measurement. For the L-treated group, the resulting solution was irradiated under 808 nm laser (50 mW cm^-2^; 1 W cm^-2^) for 5 min before detection.

**Photothermal Properties Measurement**

The photothermal conversion efficiency of STGC-PEG and GNRs-PEG was calculated according to the work reported by Roper et al [2]. DI-water (1 ml), STGC-PEG (12 μg ml^-1^ of Au, 1 ml) or GNRs-PEG (12 μg ml^-1^ of Au, 1 ml) was irradiated with 808 nm laser (1 W cm^-2^) for 20 min, respectively, to raise the temperature to a plateau, followed by a natural cooling procedure to the RT without laser irradiation, and the temperature variation was monitored on a temperature controller AI-518P (YUDIAN Automation Technology Co., Ltd., China).

**Cellular Uptake**

STGC-PEG was modified with rhodamine B isothiocyanate (RITC) for fluorescence labelling. 4T1 cells were seeded in confocal plates at 1×10^5^ cells/plate and incubated for 12 h. Then cells were treated with RITC-labelled STGC-PEG (7.5 μg ml^-1^ of Au). After several time periods (1, 4, 8 and 12 h), cells were washed with PBS for three times, stained with DAPI and Lysotracker Green, and then observed under a confocal laser scanning microscope (CLSM, Olympus FV1000, Japan) to confirm cellular uptake.

**Cell Viability Assays In Vitro**

4T1 cells were seeded in 96-well plates at 1×10^4^ cells/well and incubated for 12 h. After adhering, cells were co-incubated with STGC-PEG at different concentrations (0, 3.13, 6.25, 12.5, 25.0 and 50.0 μg ml^-1^ of Au) for 24 h. Then cells were treated with MTT solutions (5 mg ml^-1^). After incubation for 4 h, the media were replaced with DMSO and the absorbance of each well was measured at 570 nm via a microplate reader (Bio-Rad, Hercules, California, USA). In addition, cell viability of MGC-PEG-treated group was also detected using the similar methods.

For the L-treated groups, cells were treated with STGC-PEG at different concentrations (0, 3.13, 6.25, 12.5, 25.0 and 50.0 μg ml^-1^ of Au) for 8 h, and each well was washed with fresh RPMI 1640 medium and irradiated under 808 nm laser (50 mW cm^-2^) for 5 min. After incubation for 12 h, MTT assays were carried out to detect cell viability. Similarly, cell viability of MGC-PEG-treated group was also detected using the similar methods.

**Intracellular ROS Detection**

2’,7’-dichlorofluorescein diacetate (DCFH-DA) was used for the intracellular ROS detection. 4T1 cells were seeded in confocal plates at 1×10^5^ cells/plate, and treated with PBS, MGC-PEG, or STGC-PEG (25.0 μg ml^-1^ of Au) in the presence or absence of 808 nm laser (50 mW cm^-2^, 5 min), respectively. Then, the intracellular ROS levels were analyzed by a CLSM.

**JC-1 Staining**

4T1 cells were seeded in confocal plates at 1×10^5^ cells/plate, and treated with PBS, MGC-PEG, or STGC-PEG (25.0 μg ml^-1^ of Au) in the presence or absence of 808 nm laser (50 mW cm^-2^, 5 min), respectively. Then, the cells were stained with JC-1 working solution for 20 min and washed with PBS for further CLSM detection.

**Flow Cytometry Analysis**

4T1 cells were seeded in 6 well-plates at 1×10^5^ cells/plate, and treated with PBS, MGC-PEG, or STGC-PEG (25.0 μg ml^-1^ of Au) in the presence or absence of 808 nm laser (50 mW cm^-2^, 5 min), respectively. After incubation for 24 h, cells were suspended and stained with propidium iodide and FITC-Annexin V reagent for flow cytometry analysis on a FACS Calibur Flow Cytometer (Becton Dickinson, USA).

**Western Blot Analysis**

4T1 cells were treated with PBS, MGC-PEG, or STGC-PEG (25.0 μg ml^-1^ of Au) in the presence or absence of 808 nm laser (50 mW cm^-2^, 5 min). Afterwards, the cells were treated with RIPA buffer which contained protease inhibitors to extract total proteins. Proteins samples were separated with 10% SDS-PAGE gel, and then transferred to PVDF membranes. Membranes were incubated in fat-free milk at RT for 1 h, and then being incubated with the primary antibodies at 4 °C overnight. After that, membranes were incubated with secondary antibodies at RT for 2 h. The signals were detected by a chemiluminescence system (Amersham Imager 680).

**Anti-Tumor Effect In Vivo**

1×10^6^ 4T1 cells were suspended in 50 μl PBS and injected into the mammary fat pad of the 5-week female Balb/c nude mice to establish the orthotopic breast cancer mice models. When the tumors reached ~70 mm^3^, the mice were randomly divided into 6 groups (n=5) for different treatments, including PBS, MGC-PEG, STGC-PEG, PBS + L, MGC-PEG + L, and STGC-PEG + L. PBS, MGC-PEG, or STGC-PEG (1.5 mg kg^-1^) were intratumorally injected into the tumor-bearing mice in each group. And then laser treatments (50 mW cm^-2^, 5 min) was applied to trigger the photocatalytic therapy of the tumor-bearing mice. The tumor volumes and the body weights of the mice were measured every two days during the whole 14-day treatments. The tumor volume was calculated according to the formula: tumor volume = length × width^2^ / 2. Finally, the tumor slice staining including hematoxylin and eosin (H&E), terminal deoxynucleotidyl transferase dUTP nick end label (TUNEL) and ROS staining were carried out.

**Biosafety Test In Vivo**

The procedures conducted on the animals were approved by the Institutional Animal Care and Use Committee of Zhejiang University and followed the National Guidelines for Animal Protection. All animal studies were performed in compliance with relevant ethical regulations. Healthy mice were intravenously injected with STGC-PEG (10 mg kg^-1^). PBS-treated mice were set as control groups. The body weights of mice were recorded every other day. On 7 and 21 day after administration, mice were sacrificed to collect blood, and the main organs (heart, liver, spleen, lung and kidneys) of mice were also collected. The blood and main organs were used for further blood biochemical tests and histological staining, respectively.

**Statistical Analysis**

Data analysis was performed using the GraphPad Prism Software Version 5.0a (GraphPad, San Diego, California, USA). Data were presented as mean ± s.d., and represented a minimum of three independent experiments.

**Supplementary Figures**


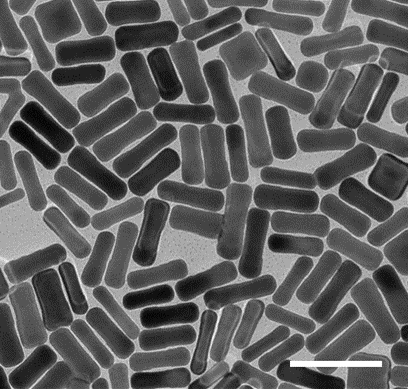


**Fig. S1** TEM image of GNRs. Scale bar, 100 nm.


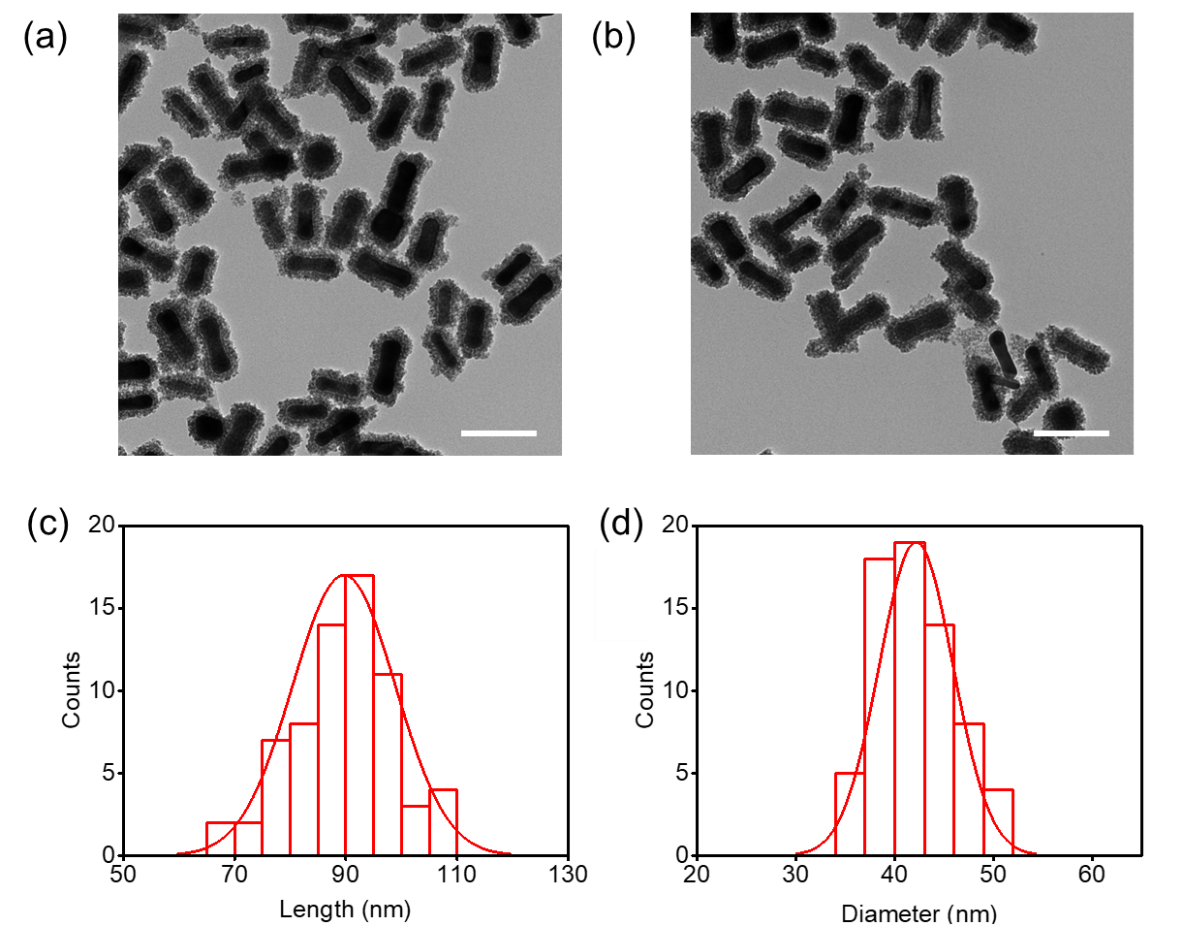


**Fig. S2** Size analysis of STGC. **a,b** The TEM images of STGC. Scale bar, 100 nm. **c,d** The length (**c**, 89.66 ± 20.95 nm) and diameter (**d**, 42.17 ± 8.56 nm) of STGC determined on TEM images (as shown in **a,b**) by using Nano Measure Software.


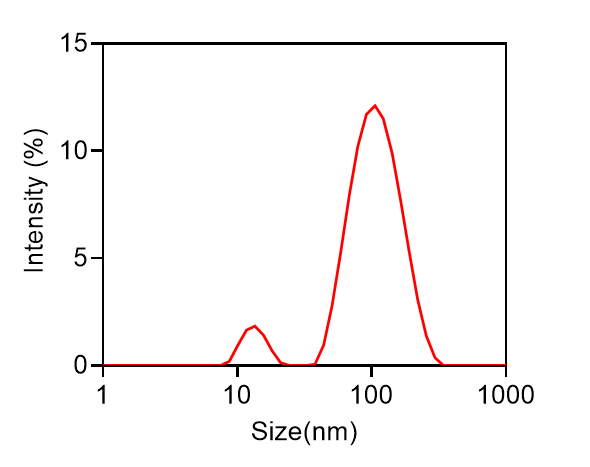


**Fig. S3** Hydrodynamic diameter distribution of STGC measured by dynamic light scattering (DLS). The average hydrodynamic diameter is ~115.3 nm.

**
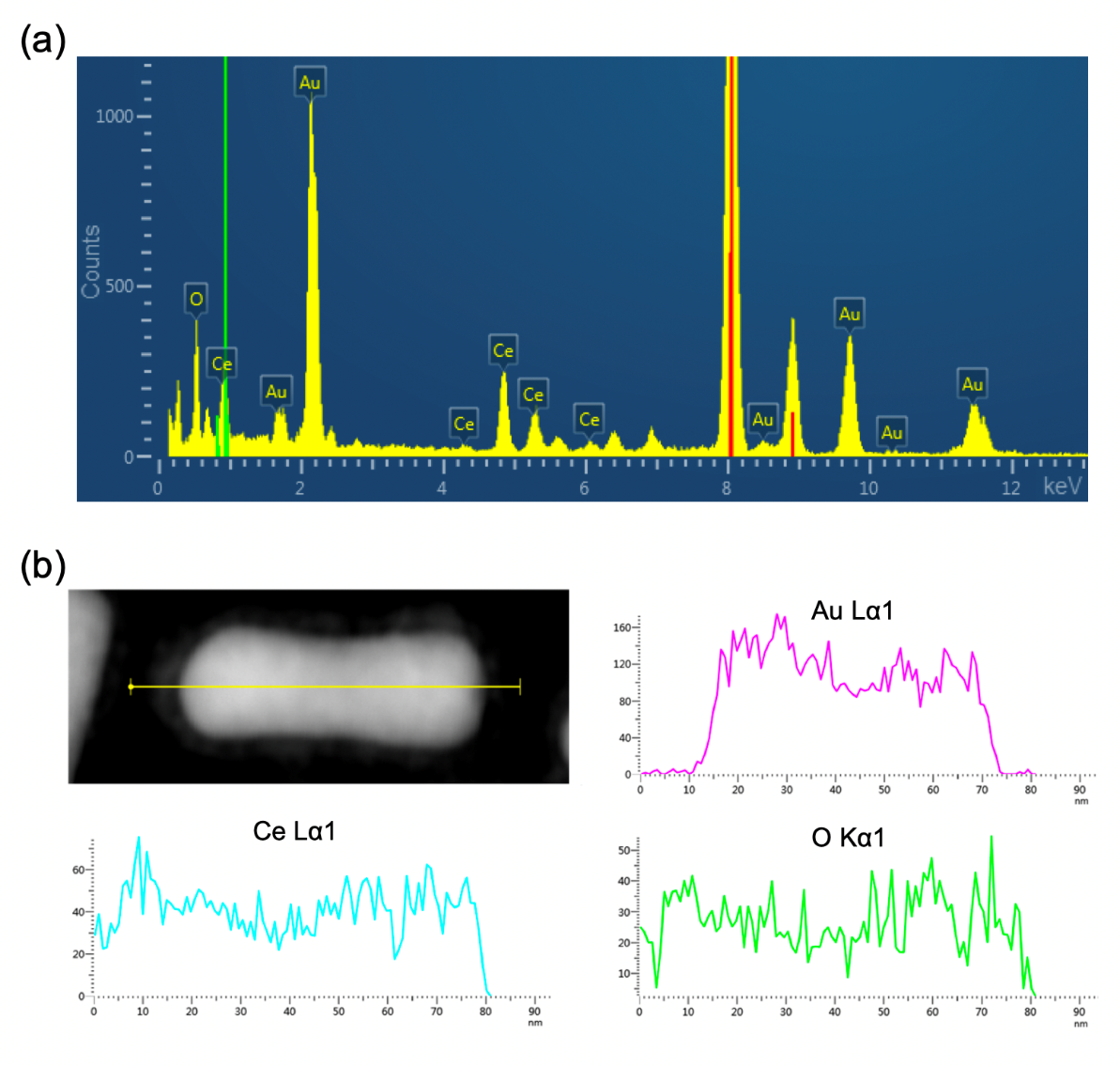
**

**Fig. S4** Characterization of STGC. **a** Energy-dispersive X-ray spectrum (EDS) of STGC. **b** HAADF-STEM image of a single STGC and the corresponding line-scanning EDS.

**Fig. S5** XPS analysis. XPS spectrum reveals the coexistence of Ce^3+^ and Ce^4+^ in STGC.

**Fig. S6** Zeta potentials of STGC and STGC-PEG. PEG coating decreases the zeta potential value of STGC from + 21.30 mV to -17.03 mV, implying the substitution of PEG for CTAB on the surface. Data are presented as mean ± s.d. (n = 3/group).


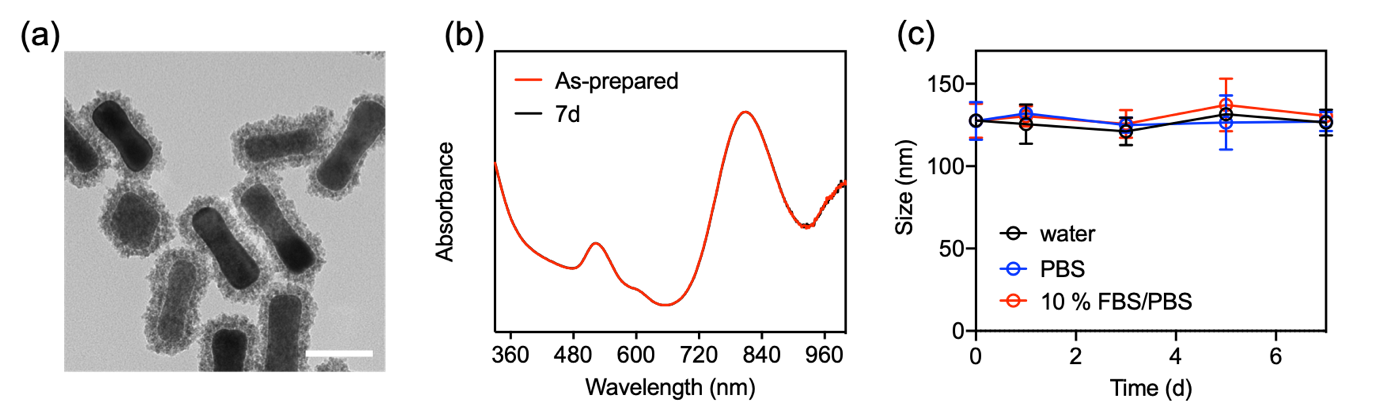


**Fig. S7** Stability tests. **a** TEM image of STGC-PEG after stored in distilled water for 7 days. Scale bar, 50 nm. **b** UV-vis absorption of STGC-PEG at 0 and 7 days after storage in distilled water. **c** Size distribution of STGC-PEG after stored in distilled water, PBS, and 10% FBS/PBS for 1, 3, 5 and 7 days. No detectable change in morphology or absorption after storage in distilled water for 7 days, indicating the excellent colloidal stability of STGC-PEG. Data are presented as means ± s.d. (n = 3/group).


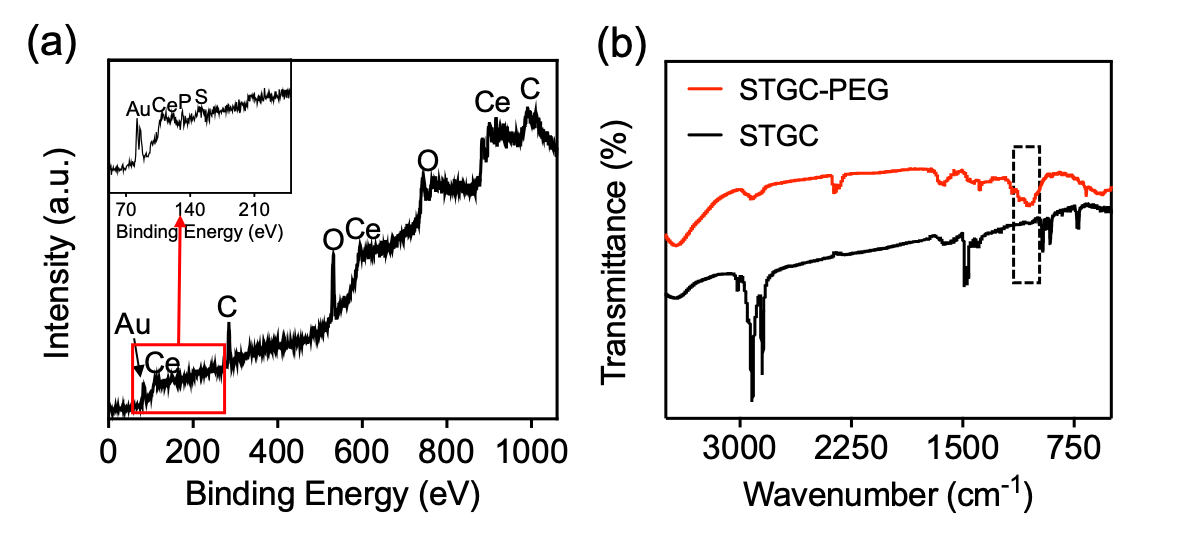


**Fig. S8** Characterization of PEG modification on STGC. **a** XPS analysis of STGC-PEG. **b** FT-IR spectra of STGC and STGC-PEG. The peaks at 133 eV and 164.05 eV confirm the existence of P and S, respectively, suggesting the successful modification of mPEG_5K_-ALN and mPEG_5K_-SH. The intensity enhancement at 1100 cm^-1^ (C-O-C stretch) in FT-IR spectra is ascribed to the amorphous PEG.

**Fig. S9** UV-vis spectra of STGC with various treatments. STGC was incubated with excess 3 % H_2_O_2_ (pH=5.0) for 12 h, which causes the redshift of absorbance peak by inducing the redox switching from Ce^3+^ to Ce^4+^ [3]. The sample was placed in a water bath (kept at RT) to avoid the photothermal effect upon NIR irradiation (808 nm, 1 W cm^-2^, 5 min).


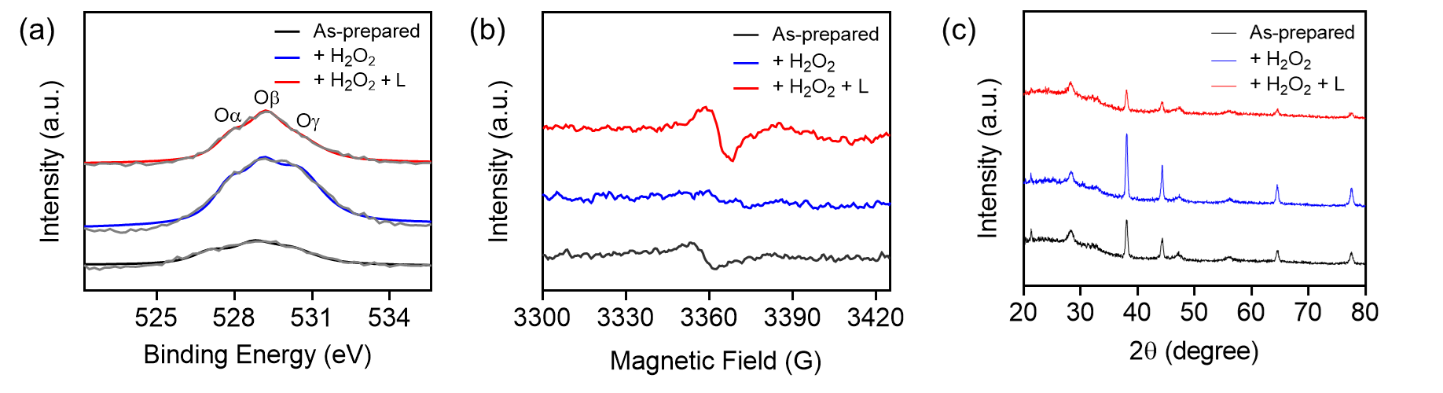


**Fig. S10** Characterization of STGC with various treatments. **a** XPS analysis of O 1s. O_β_ (O_β_ is from defective sites [4]) in STGC is calculated to be ~45.59% before treatment, which deceases to ~31.73% after incubating in H_2_O_2_, then increases to ~51.17% upon NIR irradiation. **b** ESR spectra of STGC. g=2.004. The presence of the ESR peak with the g value of 2.004 indicates the presence of OVs. The peak intensity decreases after incubation with H_2_O_2_, which recovers after NIR treatment. **c** XRD pattern of STGC.


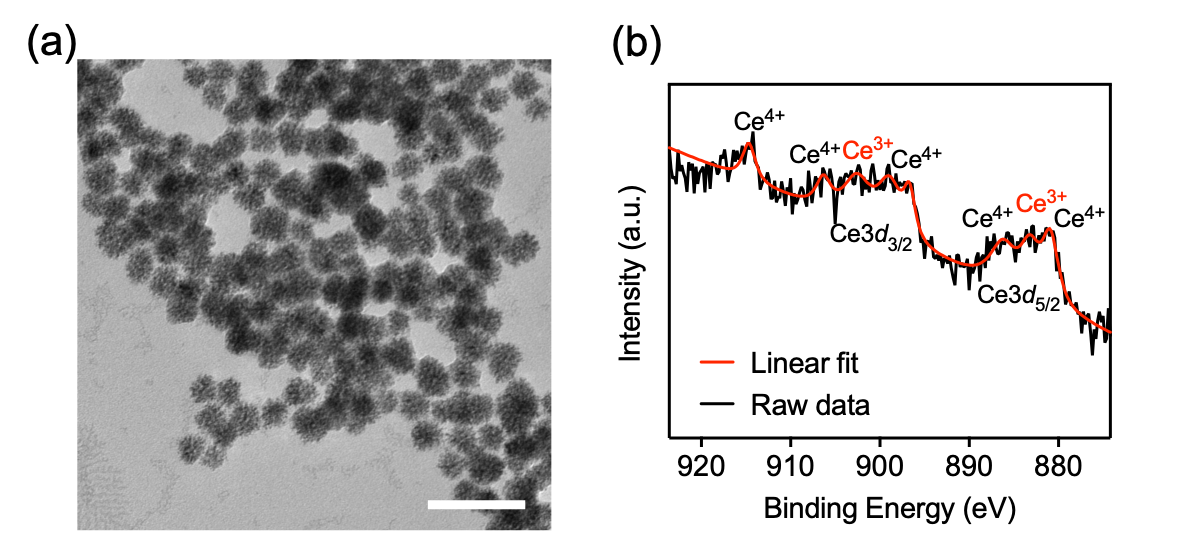


**Fig. S11** Characterization of CeO_2_ NPs. **a** TEM image of CeO_2_ NPs. Scale bar, 100 nm. **b** XPS spectrum of CeO_2_ NPs. XPS spectrum reveals the coexistence of Ce^3+^ and Ce^4+^ in CeO_2_ NPs.


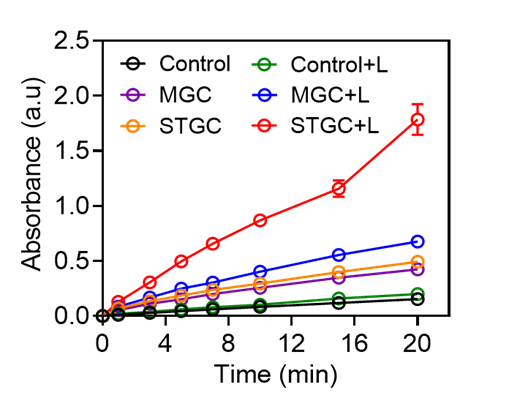


**Fig. S12** Time-course absorbance of TMB oxidation product at 650 nm upon the addition of STGC-PEG or MGC-PEG irradiated with or without 808 nm laser (1 W cm^-2^) in the presence of H_2_O_2_ (10 mM). STGC denotes STGC-PEG, MGC denotes MGC-PEG. Data are presented as means ± s.d. (n = 3/group).


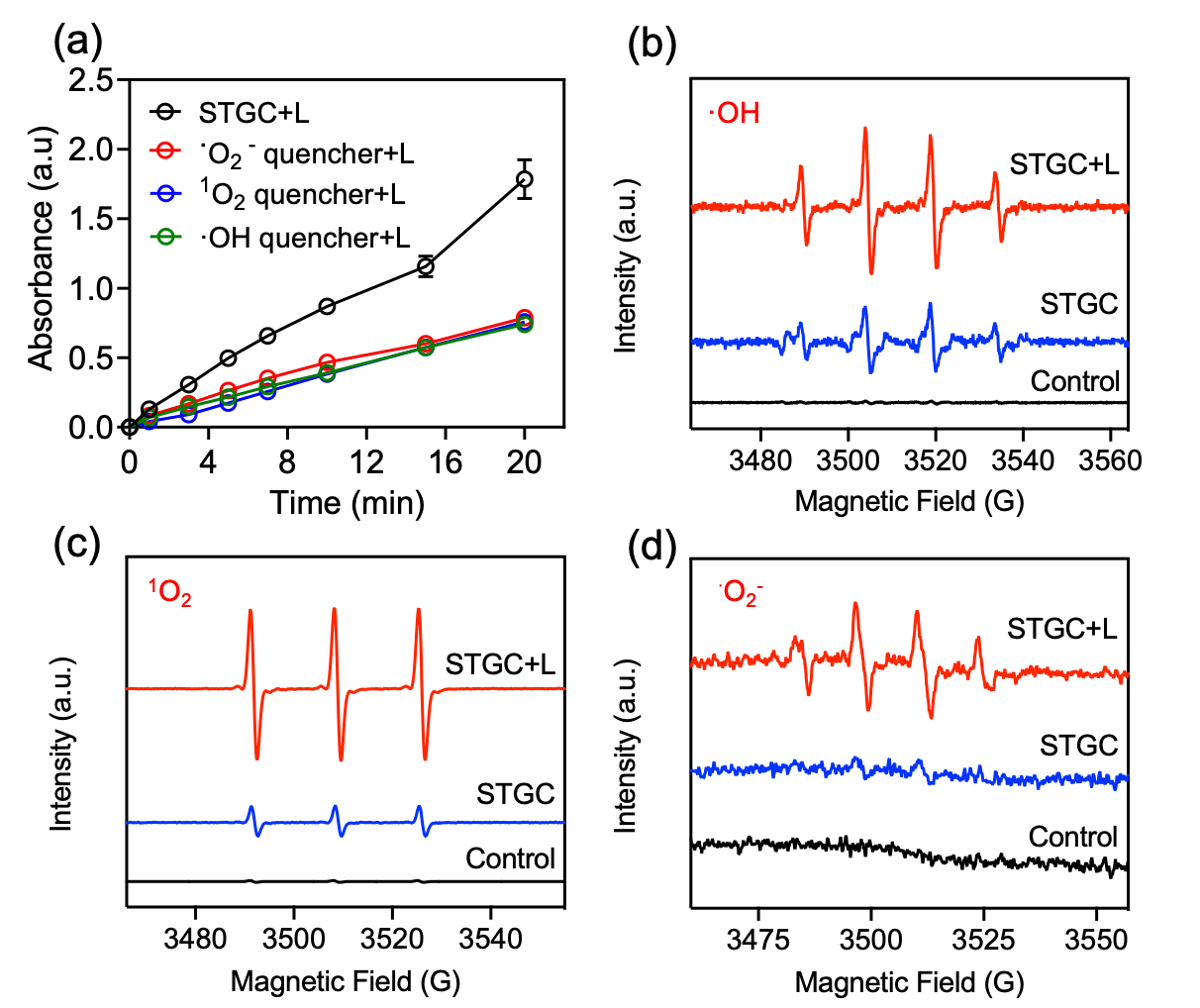


**Fig. S13** The photon-augmented ROS generation by STGC-PEG as photon-driven sub-nanostructural transformable nanozymes. **a** Time-course absorbance of TMB oxidation products at 650 nm with different scavengers with 808 nm laser (1 W cm^-2^). STGC denotes STGC-PEG. Data are presented as means ± s.d. (n = 3/group). **b** ESR spectra of DMPO-·OH spin adducts generated by STGC-PEG with or without 808 nm laser irradiation (1 W cm^-2^, 5 min) in the presence of H_2_O_2_ (10 mM). **c,d** ESR spectra of TEMP-^1^O_2_ (**c**), and BMPO-˙O_2_^−^ (**d**) spin adducts generated by STGC-PEG with or without 808 nm laser irradiation (1 W cm^-2^, 5 min).


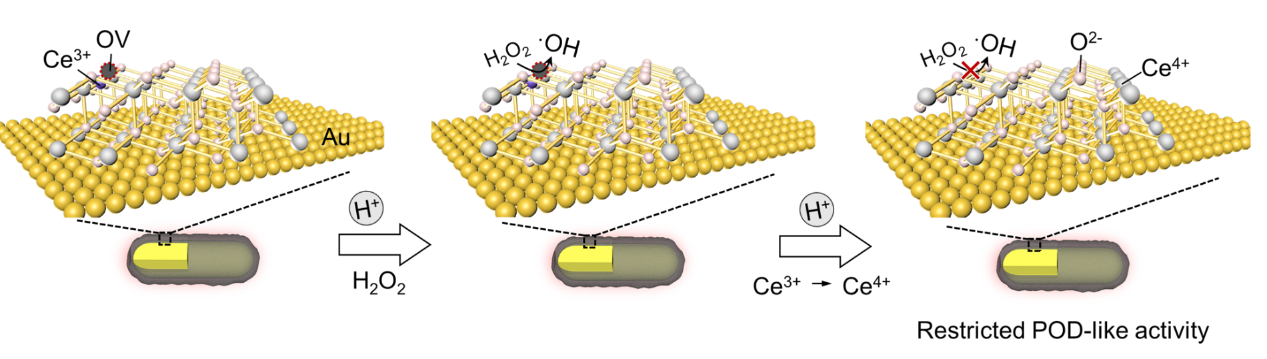


**Fig. S14** Schematic illustration of the enzymatic activity of STGC-PEG without NIR irradiation. The STGC-PEG exhibits the restricted peroxidase (POD)-like catalytic activity without NIR irradiation, since extra H^+^ retards the reduction of Ce^4+^ and thus inhibits the decomposition of absorbed H_2_O_2_.


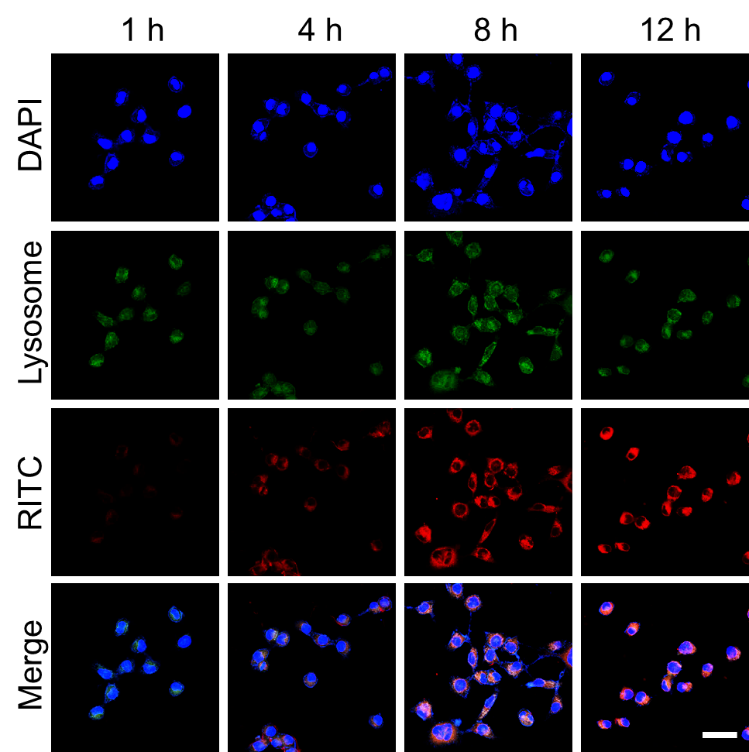


**Fig. S15** CLSM images of cellular uptake after treatment with RITC-labelled STGC-PEG. Scale bar, 40 µm.

**Fig. S16** The concentration of Au in 4T1 cells after incubation with STGC-PEG at different time points. Data are presented as mean ± s.d. (n = 4/group).


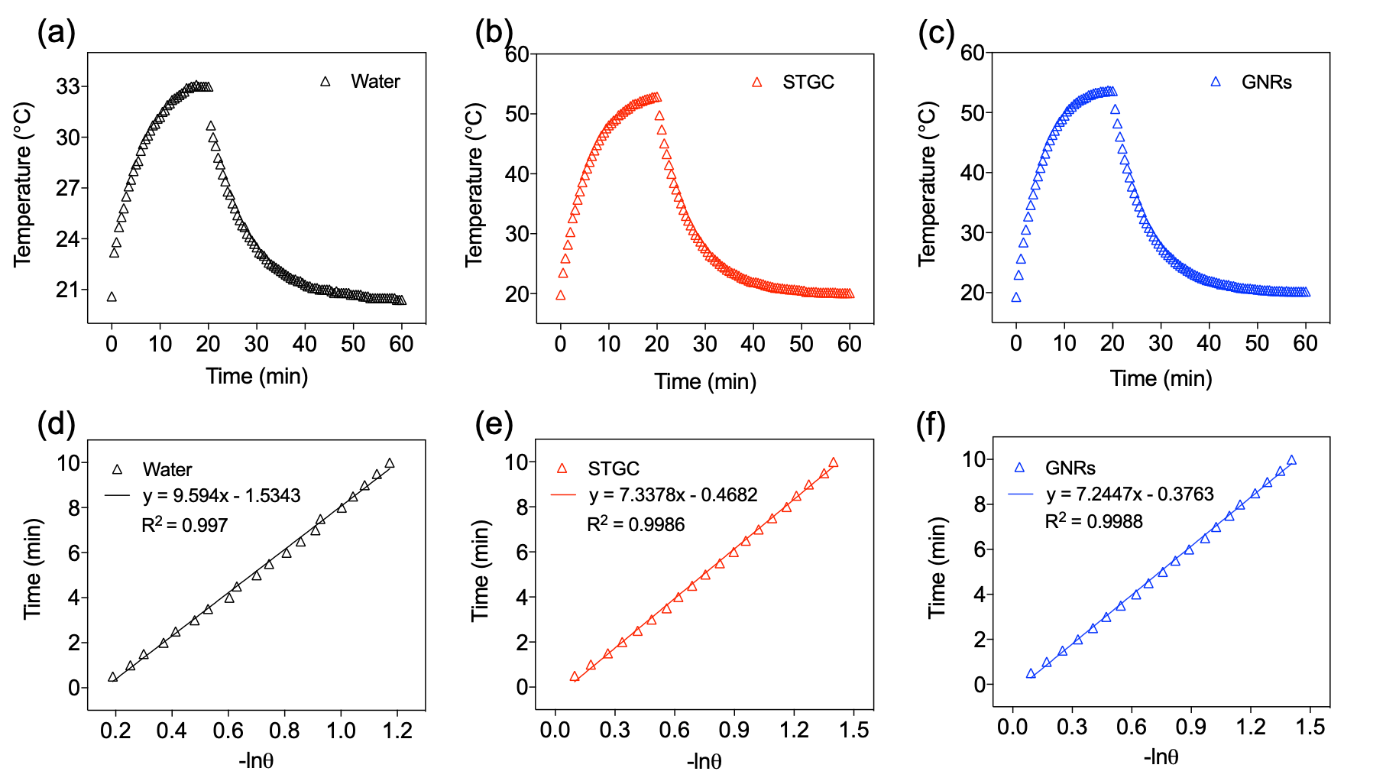


**Fig. S17** Photothermal conversion efficiency (𝜂) evaluation. **a-c** Temperature variation curves of water (**a**), STGC-PEG (**b**), and GNRs-PEG (**c**) under NIR irradiation (808 nm, 1 W cm^-2^) for 20 min, followed by natural cooling without NIR irradiation. STGC denotes STGC-PEG, GNRs denotes GNRs-PEG. **d-f** Determination of time constant by linear fitting of the cooling period of water (**d**), STGC-PEG (**e**), and GNRs-PEG (**f**).


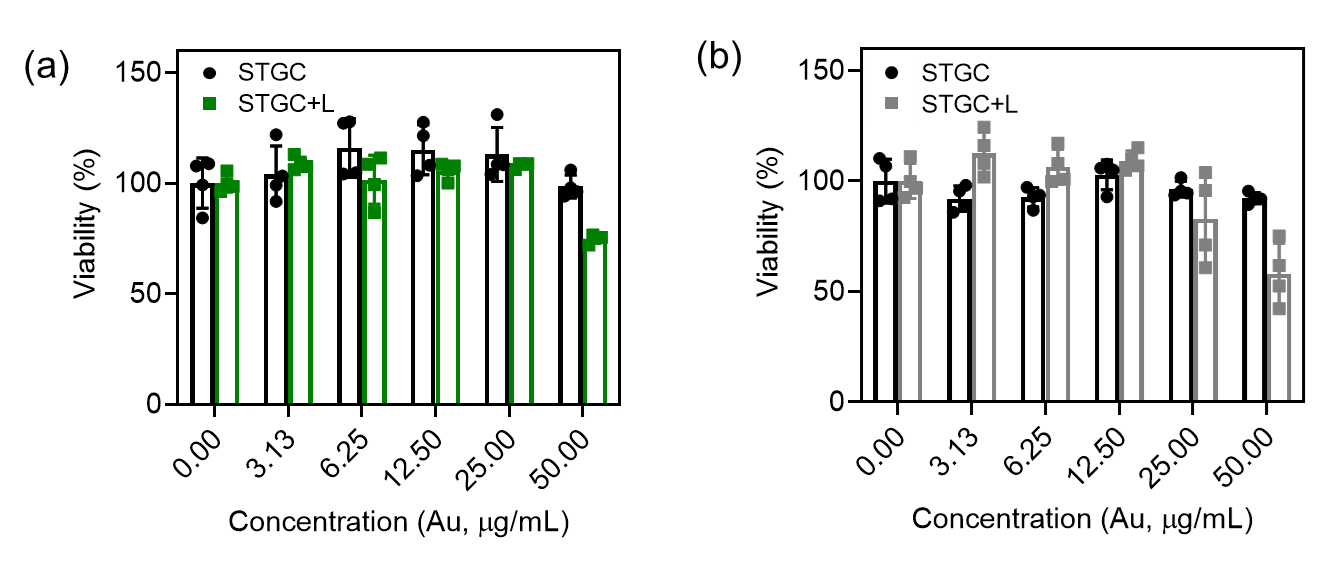


**Fig. S18** In vitro cellular toxicity profiles of STGC-PEG on human normal cell lines. **a,b** Cellular toxicity profile of STGC-PEG on hepatic cell L02 (**a**) and colonic epithelial cell NCM460 (**b**) without or with 808 nm laser irradiation (50 mW cm^-2^, 5 min). STGC denotes STGC-PEG. Data are presented as means ± s.d. (n = 4/group).

**Fig. S19** Tumor weight of 4T1 tumor-bearing mice with different treatments. Data are presented as mean ± s.d. (n = 5/group). STGC denotes STGC-PEG, MGC denotes MGC-PEG.

**Fig. S20** Body weight of 4T1 tumor-bearing mice with different treatments. Data are presented as mean ± s.d. (n = 5/group). STGC denotes STGC-PEG, MGC denotes MGC-PEG.


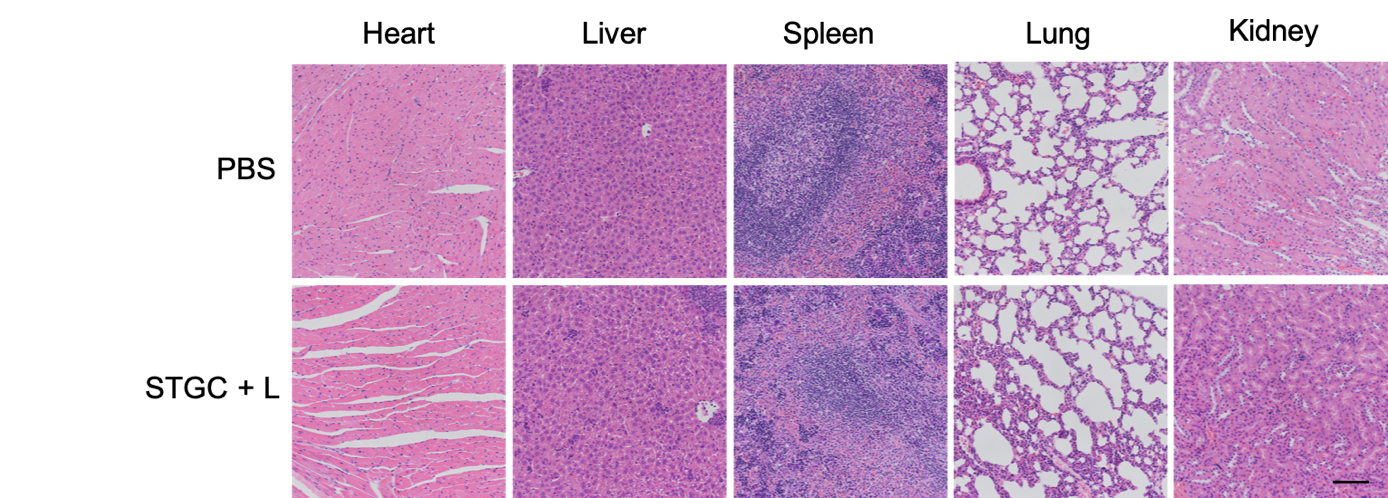


**Fig. S21** H&E staining of main organs (heart, liver, spleen, lung, and kidney) of tumor-bearing mice with different treatments. Scale bar, 200 µm. STGC denotes STGC-PEG.

**Fig. S22** Body weight changes of mice after intravenous injection of PBS or STGC-PEG. Data are presented as mean ± s.d. (n = 4/group). STGC denotes STGC-PEG.


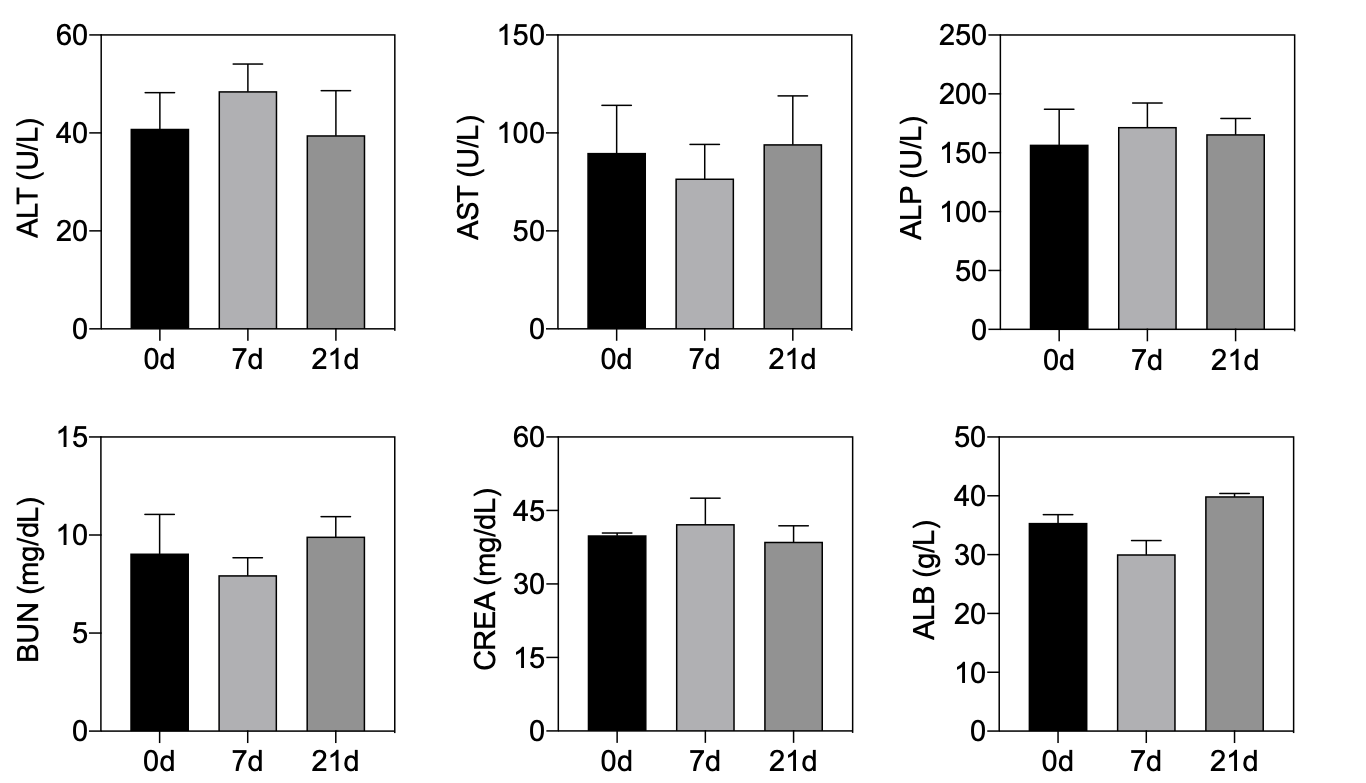


**Fig. S23** Biochemical analysis of mice on 0, 7 and 21 day after intravenous injection of PBS or STGC-PEG. Data are presented as mean ± s.d. (n = 4/group).


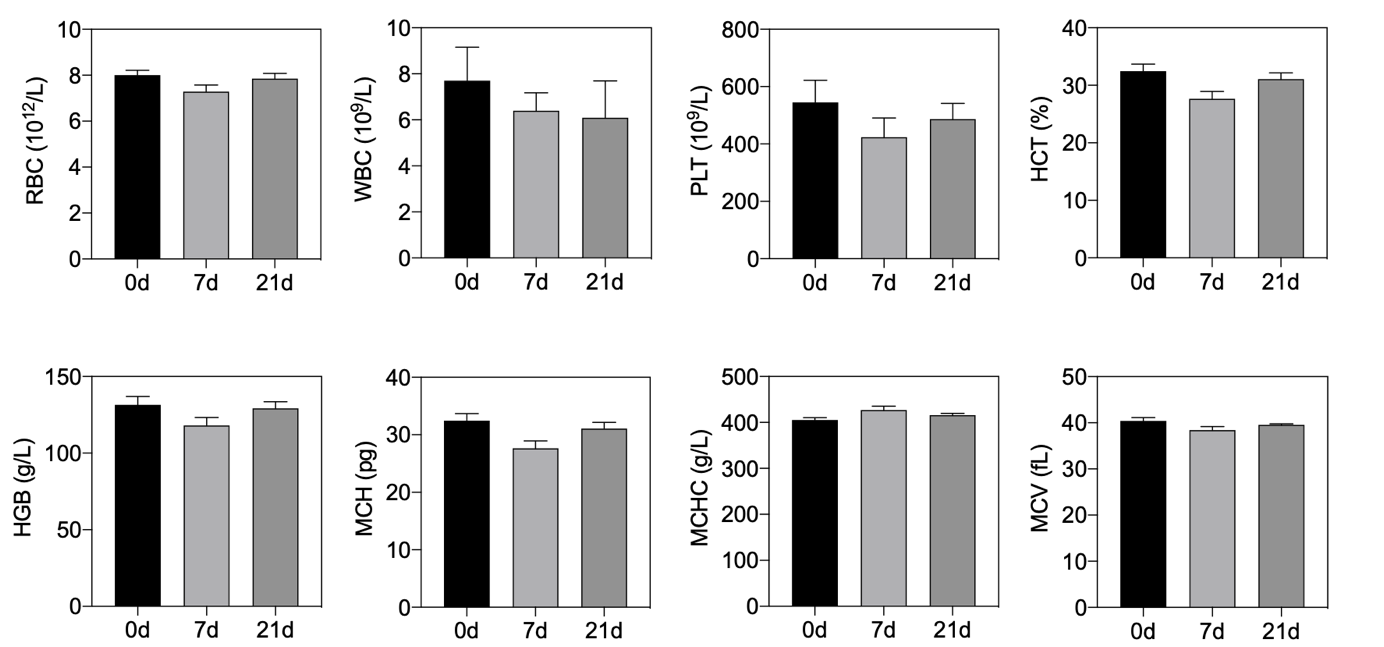


**Fig. S24** Blood analysis of mice on 0, 7 and 21 day after intravenous injection of PBS or STGC-PEG. Data are presented as mean ± s.d. (n = 4/group).


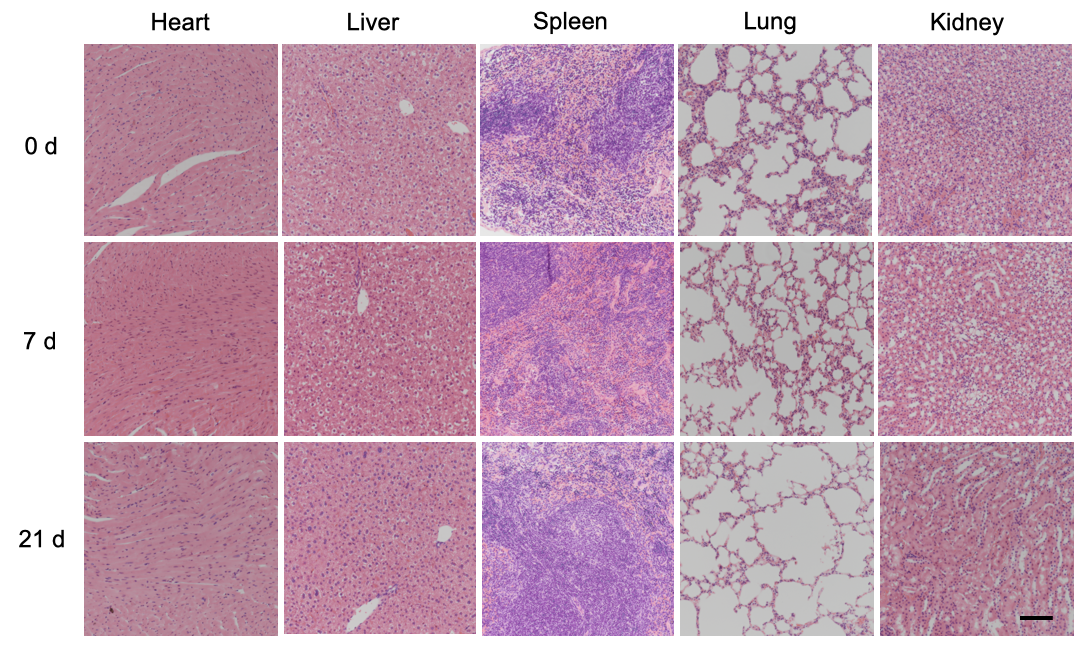


**Fig. S25** Hematoxylin and eosin (H&E) staining of main organs (heart, liver, spleen, lung, and kidney) of mice on 0, 7 and 21 day after intravenous injection of PBS or STGC-PEG. STGC denotes STGC-PEG. Scale bar, 500 µm.

**Supplementary References**

[1] R. Long, K. Mao, X. Ye, W. Yan, Y. Huang et al. Surface facet of palladium nanocrystals: a key parameter to the activation of molecular oxygen for organic catalysis and cancer treatment. J. Am. Chem. Soc. **135**(8), 3200-3207 (2013). https://doi.org/10.1021/ja311739v

[2] Y. Zhou, Q. Xu, T. Ge, X. Zheng, L. Zhang et al. Accurate control of VS_2_ nanosheets for coexisting high photoluminescence and photothermal conversion efficiency. Angew. Chem. Int. Edit. **59**(8), 3322-3328 (2020). <https://doi.org/10.1002/anie.201912756>

[3] B. Li, T. Gu, T. Ming, J. Wang, P. Wang et al. (Gold core)@(ceria shell) nanostructures for plasmon-enhanced catalytic reactions under visible light. ACS Nano **8**(8), 8152-8162 (2014). <https://doi.org/10.1021/nn502303h>

[4] W, Guo, M. Zhang, Z. Lou, M. Zhou, P. Wang et al. Engineering nanoceria for enhanced peroxidase mimics: a solid solution strategy. ChemCatChem. **11**(2), 737-743 (2019). https://doi.org/10.1002/cctc.201801578
